# Validation of the Skåne University Hospital nomogram for the preoperative prediction of a disease-free axilla in patients with breast cancer

**DOI:** 10.1093/bjsopen/zrab027

**Published:** 2021-06-22

**Authors:** S Majid, P-O Bendahl, L Huss, J Manjer, L Rydén, L Dihge

**Affiliations:** Department of Clinical Sciences Malmö, Lund University, Malmö, Sweden; Department of Surgery, Skåne University Hospital, Lund-Malmö, Sweden; Department of Oncology and Pathology, Clinical Sciences, Lund University, Sweden; Department of Clinical Sciences Malmö, Lund University, Malmö, Sweden; Department of Surgery, Helsingborg Hospital, Helsingborg, Sweden; Department of Clinical Sciences Malmö, Lund University, Malmö, Sweden; Department of Surgery, Skåne University Hospital, Lund-Malmö, Sweden; Department of Surgery, Skåne University Hospital, Lund-Malmö, Sweden; Department of Clinical Sciences Lund, Lund University, Lund, Sweden; Department of Clinical Sciences Lund, Lund University, Lund, Sweden; Department of Plastic and Reconstructive Surgery, Skåne University Hospital, Malmö, Sweden

## Abstract

**Background:**

Axillary staging via sentinel lymph node biopsy (SLNB) is performed for clinically node-negative (N0) breast cancer patients. The Skåne University Hospital (SUS) nomogram was developed to assess the possibility of omitting SLNB for patients with a low risk of nodal metastasis. Area under the receiver operating characteristic curve (AUC) was 0.74. The aim was to validate the SUS nomogram using only routinely collected data from the Swedish National Quality Registry for Breast Cancer at two breast cancer centres during different time periods.

**Method:**

This retrospective study included patients with primary breast cancer who were treated at centres in Lund and Malmö during 2008–2013. Clinicopathological predictors in the SUS nomogram were age, mode of detection, tumour size, multifocality, lymphovascular invasion and surrogate molecular subtype. Multiple imputation was used for missing data. Validation performance was assessed using AUC and calibration.

**Results:**

The study included 2939 patients (1318 patients treated in Lund and 1621 treated in Malmö). Node-positive disease was detected in 1008 patients. The overall validation AUC was 0.74 (Lund cohort AUC: 0.75, Malmö cohort AUC: 0.73), and the calibration was satisfactory. Accepting a false-negative rate of 5 per cent for predicting N0, a possible SLNB reduction rate of 15 per cent was obtained in the overall cohort.

**Conclusion:**

The SUS nomogram provided acceptable power for predicting a disease-free axilla in the validation cohort. This tool may assist surgeons in identifying and counselling patients with a low risk of nodal metastasis on the omission of SLNB staging.

## Teaser

The Skåne University Hospital nomogram was developed to assess the possibility of omitting sentinel lymph node biopsy (SLNB) for breast cancer patients with a low risk of nodal metastasis. The nomogram provided acceptable power for predicting node-negative disease in validation cohort. Accepting false negative rates of 5-10 per cent for predicting disease-free axilla, possible SLNB reduction rates in the range of 15–26 per cent were obtained.

## Introduction

Assessment of patients’ axillary lymph node (ALN) status and the number of metastatic lymph nodes is essential for planning the treatment of primary breast cancer and underscores the importance of accurate nodal staging. Nodal staging via sentinel lymph node biopsy (SLNB) is routinely performed for all patients with a clinically negative axilla. The oncological safety of SLNB has been widely documented, even at a false-negative rate (FNR) of 5–10 per cent^1–3^. However, early detection via public mammography screening programmes has decreased the rate of node-positive disease^4^. Thus, invasive staging typically reveals a disease-free axilla (N0) in most primary breast cancer patients, and SLNB yields no therapeutic benefit. Moreover, although SLNB is a minimally invasive procedure and is associated with significantly less postoperative morbidity than axillary lymph node dissection (ALND), surgical axillary staging results in an incidence rate of 5–7 per cent for lymphoedema, 11 per cent for arm pain/numbness and 23 per cent for quality-of-life impairment^5^.

The benefit of extensive nodal staging is debatable, as advances in adjuvant therapy are tailored to tumour features rather than pathological nodal status^6^^,^^7^. The need for complete ALND when SLNB confirms limited disease has been questioned based on the lack of adverse effects on survival when ALND was omitted during randomized trials, such as the IBCSG 23–01 trial^5^ and the ACOSOG Z0011 trial^6^ (with a 10-year median follow-up period). There is also a growing interest in the reduction of axillary surgical staging and SLNB omission for selected low-risk patients based on the ongoing prospective European SOUND^8^ and INSEMA^9^ randomized trials. In these trials, breast cancer patients who are candidates for breast-conserving surgery with clinically and ultrasonographically negative axillae are randomized to undergo SLNB or no surgical axillary staging. Moreover, physical examination of the axilla is a poor predictor of ALN metastasis, with a sensitivity of approximately 30 per cent^10^^,^^11^. Axillary ultrasonography is also an unreliable preoperative staging modality for patients with a low nodal metastatic burden^12^^,^^13^, with a pooled estimated sensitivity of 50 per cent and an FNR of 25 per cent^14^. Although improvements in imaging technologies are promising, their accuracy remains inferior to that of surgical staging, and imaging modalities alone cannot replace SLNB for nodal staging^15^.

**Fig. 1:**
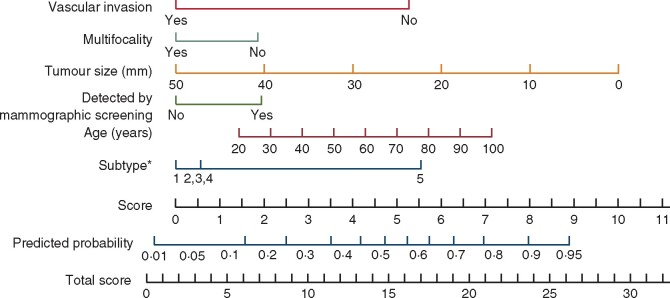
The Skåne University Hospital nomogram for predicting a disease-free axilla *versus* any nodal metastasis The total score for each patient is assigned by drawing a vertical line from the appropriate point for each predictor down to the score scale and summing these scores. The predicted probability of N0 is determined by drawing a vertical line from the total score scale up to the predicted probability scale in the lower part of the nomogram. *Subtypes: 1, luminal A-like; 2, luminal B-like (LumB)/human epidermal growth factor receptor 2 (HER2)-negative; 3, LumB/HER2-positive; 4, HER2-positive/non-luminal; 5, triple-negative. Copyright^©^2017 Wiley, used with permission from: Dihge L, Bendahl PO, Ryden L. Nomograms for preoperative prediction of axillary nodal status in breast cancer. *Br J Surg* 2017;**104**:1494–1505

Predictive models based on clinical and histopathological features have been developed to improve the prediction of axillary nodal status. One of the first nomograms for estimating the likelihood of a positive SLNB result was developed in 2007 at the Memorial Sloan-Kettering Cancer Center, which provided an area under the receiver operating characteristic curve (AUC) value of 0.75^16^. Although the accuracies of predictive models have been confirmed as being satisfactory, their predictive abilities are often reduced outside the centre in which they were initially developed^17^^,^^18^. Thus, prediction of axillary nodal spread based on clinicopathological variables is considered imperfect and consequently SLNB remains the standard ALN staging procedure. The research group at Skåne University Hospital (SUS) has evaluated whether the preoperative tumour detection mode and clinicopathological determinants could be used to predict lymphatic spread. In agreement with previous publications, the triple-negative molecular subtype, which is associated with worse prognosis, was shown to metastasize infrequently to the ALNs^19^^,^^20^. Based on those results, the SUS nomogram was proposed in 2017 for predicting a disease-free axilla (N0 *versus* N+)^21^ which could help surgeons identify patients with a low risk of any nodal metastasis using six determinants (age, mode of detection, tumour size, multifocality, vascular invasion and surrogate molecular subtype of the breast cancer) (*Fig. 1*). Internal validation revealed good discrimination with an AUC value of 0.74 (95 per cent c.i. 0.70 to 0.79).

The present study aimed to validate the SUS nomogram in a population-based cohort from breast cancer centres in Lund and Malmö, using only routinely collected data from The National Quality Registry for Breast Cancer (NKBC) of Sweden^22^. This study also aimed to determine the per cent reduction in unbeneficial SLNB that could be achieved using the SUS nomogram to identify patients with the lowest risk of nodal metastasis, who might be spared from surgical axillary staging.

## Methods

The retrospective study protocol was approved by the regional ethical review board (Lund, Sweden; Dnr 2013/821). All model validation procedures are reported in accordance with the EQUATOR guidelines for the transparent reporting of diagnostic studies, the TRIPOD statement^23^ and the STARD 2015 checklist^24^.

### Validation cohort

This validation study included patients with primary invasive breast cancer who underwent breast surgery and axillary staging at two breast cancer centres in Lund and Malmö (Skåne University Hospital) between January 2008 and December 2013. The validation cohort was stratified according to site (Lund and Malmö) and time period: overlapping the development period for the original SUS nomogram (2009–2012);1 year before and 1 year after the nomogram development period (2008 and 2013); and the entire validation period (2008–2013), which corresponded to seven separate validation analyses. As in the original report regarding the development and internal validation of the SUS nomogram, the exclusion criteria were as follows: male sex, presence of bilateral tumours, history of invasive breast cancer or *in situ* ductal carcinoma, and neoadjuvant chemotherapy. Patients with missing information regarding ALN status or the procedure for surgical axillary nodal staging were also excluded. In accordance with the Swedish National Guidelines for Breast Cancer^25^ at the time, ALND was recommended if SLNB revealed micrometastasis or macrometastasis.

### Data collection and definition of clinicopathological determinants

Data regarding the clinicopathological determinants for predicting a disease-free axilla (N0 *versus* N+) using the SUS nomogram were retrieved from the NKBC, which covers over 99 per cent of all new breast cancer cases in Sweden^26^. The determinants included in the nomogram were as follows: age, mode of detection, tumour size, multifocality, vascular invasion and St Gallen surrogate molecular subtype (*Fig. 1*). In addition, data were collected regarding baseline variables, such as tumour histopathological grade, ALN status and the axillary staging procedure. Pathological predictors included Ki-67 status, HER2 status, oestrogen receptor (ER) status, progesterone receptor (PR) status, multifocality and vascular invasion, which were defined according to the Swedish Society of Pathology classification system^27^. Positive nodes (N+) were defined as ALNs with macrometastasis or micrometastasis, and negative nodes (N0) were defined as nodes with isolated tumour cells or no metastasis. Vascular invasion was defined as the presence of tumour cell invasion through a vessel wall and endothelium or was defined based on the presence of tumour cells in vascular spaces or in the underlying endothelium of vascular channels. Multifocality was defined as the presence of two or more tumours in the same breast, which were at least 20 mm apart and separated by normal tissue or an *in situ* carcinoma.

The surrogate molecular subtype categories in the SUS nomogram were based on the proposed classification from the 13^th^ St Gallen International Breast Cancer Conference in 2013^28^, which defined five different subtypes based on the ER, PR, HER2 and Ki-67 statuses. The validation analyses defined hormone-receptor status based on ER status alone, and the Ki-67 expression level was defined as high if it was greater than 20 per cent. The surrogate molecular subtypes were defined as follows: luminal A-like (LumA: ER+, HER2–, Ki-67 low), luminal B-like/HER2 negative (LumB/HER2–: ER+, HER2–, Ki-67 high), luminal B-like/HER2 positive (LumB/HER2+: ER+, HER2+, any Ki-67), HER2-positive/non-luminal (HER2+/non‐luminal: ER–, HER2+, any Ki-67) and triple-negative (TNBC: ER–, HER2–, any Ki-67).

### Statistical analysis

Multiple imputation was applied to handle missing data regarding the determinants for the SUS nomogram. The following variables were included in the imputation models:

- The dichotomous outcome (N0 *versus* N+)
- All determinants for the SUS nomogram, except for the surrogate molecular subtype (i.e., age, tumour size, mode of detection, vascular invasion and multifocality)
- The individual components of the surrogate molecular subtype (i.e., ER status, HER2 status and Ki-67 status)
- Other potential predictors of missingness or the missing values themselves (e.g., menopausal status, histological grade, PR status, treating centre, date of diagnosis and date of surgery).

The patterns of missing data were investigated and used in the specification of the imputation models. Two hundred new complete imputed data sets were generated, and 20 iterations were used to create each of the imputed datasets. Continuous variables were imputed using linear regression, and categorical variables were imputed using predictive mean matching. Classification into surrogate molecular subtypes was performed following imputation of the individual components.

The performance of the nomogram for discriminating between N0 and N+ was analysed using a receiver operating characteristics (ROC) analysis and by summarizing the AUC values averaged over the 200 imputed datasets. The value of the linear predictor (LP), which is the predicted log odds of lymph node negativity for patients in the validation cohort when applying the weights from the logistic regression model underlying the SUS nomogram, was used in the ROC analysis:

$$\begin{array}{c} \text{LP}=-1.793+0.166\times \left(\text{LumB}/\text{HER}2-\right)+0.102\times \left(\text{LumB}/\text{HER}2+\right)+0.389\\ \times \left(\text{HER}2+/\text{non}-\text{luminal}\right)+1.620\times \left(\text{TNBC}\right)+0.021\times \left(\text{age}\;\text{in}\;\text{years}\right)+\\ 0.562\times \left(\text{screening}\;\text{detected}\right)-0.059\times \left(\text{tumour}\;\text{size}\;\text{in}\;\text{mm}\right)+0.542\times (\text{no}\\ \text{multifocality})+1.542\times \left(\text{no}\;\text{vascular}\;\text{invasion}\right) \end{array}$$

The first four variables in the equation are dummy variables comparing the subtype in the parentheses (coded 1) to the luminal A-like subtype as the reference subtype (coded 0). In addition, three binary predictors (screening detected disease, no multifocality and no vascular invasion) are coded 1 for logical yes and 0 for logical no.

The inverse logit transformation was subsequently used to calculate the predicted probability of N0:

p= exp (LP)/[1+ exp (LP)]

A pooled AUC was calculated as the mean of the 200 imputation-specific AUC estimates. The variance of this estimate, which is the sum of the variances within and between imputations, was calculated using Rubin’s rule. The Hosmer–Lemeshow test and the corresponding calibration plot were applied to evaluate the goodness of fit of the logistic regression model underlying the SUS nomogram in the validation dataset. The predicted probabilities (p) of N0 were divided into deciles, and the observed proportions of patients with a disease-free axilla were compared to the average predicted probabilities in each decile. Thus, 10 dots on a line with a 45° slope would reflect perfect calibration. The calibration plots for the present study have 200 dots per decile, which correspond to one dot per imputed dataset.

The cut-off point for predicting a disease-free axilla was set at the maximized negative predictive value (NPV, the NPV closest to 100 per cent), which would identify patients with a very low probability of nodal metastasis who would not be likely to benefit from surgical axillary staging via SLNB. The mean values from the 200 imputed datasets, which were rounded to the nearest integer, for true-positive (TP), true-negative (TN), false-positive (FP), and false-negative (FN) results were calculated and used to calculate the FNR as FN/(FN + TP). The possible SLNB reduction rates were defined as (TN + FN)/(TN + FN + TP + FP), based on a maximized NPV and maximum FNRs of 5 per cent and 10 per cent to reflect the acknowledged FNRs for SLNB staging. All calculations were performed using IBM SPSS^®^ software (version 25.0; IBM Corp., Armonk, New York, USA) and Stata^®^ software (version 16.0; StataCorp, College Station, Texas, USA).

## Results

**Fig. 2:**
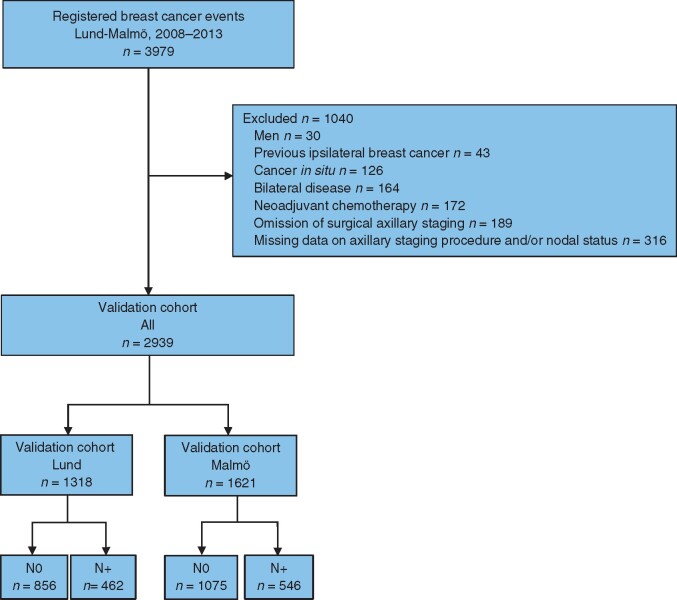
Flow chart for the validation cohorts N0, disease-free axilla; N+, any lymph node metastasis

Between January 2008 and December 2013, 3979 patients were diagnosed with breast malignancy and scheduled for treatment at the SUS centres in Lund and Malmö. Patients were excluded if they were of male sex (30 patients), had a history of invasive breast cancer (43) or *in situ* carcinoma (126), were confirmed to have received neoadjuvant treatment (170), any uncertainty regarding whether neoadjuvant therapy was administered (2), or missing information regarding the procedure for axillary nodal staging (189) or the final nodal status (316). Thus, the final study population included 2939 eligible patients: 1318 patients who were treated in Lund and 1621 patients who were treated in Malmö (*Fig. 2*). The clinicopathological characteristics and tumour detection modes for the overall validation cohort, the Lund centre and the Malmö centre are shown in *Table 1*.

**Table 1:** Comparison of baseline patient and tumour characteristics in the validation cohort

	**All (Lund and Malmö)** **2008–2013**	**Lund** **2008–2013**	**Malmö** **2008–2013**	***P*** ‡ **Lund *versus* Malmö**
**No. (row percentage)**	2939	1318 (45)	1621 (55)	
Age (years)*	62 (24–96)	62 (24–91)	62 (29–96)	0.664§
**Mode of detection**				<0.001
Mammography screening	1518 (53)	734 (58)	784 (48)	
Symptomatic presentation	1360 (47)	523 (42)	837 (52)	
Missing	61	61	0	
**Tumour size (pT)†**				0.004¶
≤20 mm (pT1)	1877 (67)	840 (70)	1037 (65)	
>20–≤50 mm (pT2)	860 (31)	338 (28)	522 (33)	
>50 mm (pT3)	61 (2)	22 (2)	39 (2)	
Missing	141	118	23	
**Histological grade**				0.258¶
I	649 (22)	281 (22)	368 (23)	
II	1258 (43)	556 (43)	702 (44)	
III	993 (34)	457 (35)	536 (33)	
Missing	39	24	15	
**Multifocality**				0.956
Absent	1804 (79)	679 (79)	1125 (79)	
Present	480 (21)	180 (21)	300 (21)	
Missing	655	459	196	
**Vascular invasion**				<0.001
Absent	1565 (85)	194 (76)	1371(86)	
Present	280 (15)	62 (24)	218 (14)	
Missing	1094	1062	32	
**Oestrogen receptor**				0.199
Positive	2444 (87)	1062 (88)	1382 (86)	
Negative	366 (13)	146 (12)	220 (14)	
Missing	129	110	19	
**Progesterone receptor**				0.828
Positive	2088 (74)	900 (75)	1188 (74)	
Negative	720 (26)	307 (25)	413 (26)	
Missing	131	111	20	
**HER2 status**				<0.001
Not amplified	1587 (84)	955 (88)	632 (78)	
Amplified	312 (16)	129 (11)	183 (23)	
Missing	1040	234	806	
**Ki-67**				0.023
≤20%	232 (50)	93 (57)	139 (46)	
>20%	233 (50)	70 (43)	163 (54)	
Missing	2474	1155	1319	
**Regional lymph node metastases**				0.436
N0	1931 (66)	856 (65)	1075 (66)	
N+	1008 (34)	462 (35)	546 (34)	

Values in parentheses are column percentages unless indicated otherwise;

* median (range).

† According to the TNM classification for breast cancer. N0, disease-free axilla;

N+, any lymph node metastasis.

‡ Pearson's

χ2 test, except

§ Mann–Whitney *U* test;

¶ χ2 test for trend.

### Validation cohort characteristics

The axillary staging procedure involved SLNB for 1801 patients (61.3 per cent), nodal sampling for 18 patients (0.6 per cent) and ALND for 1115 patients (37.9 per cent). Axillary metastasis was detected in 1008 patients (34 per cent) in the overall validation cohort (*Table 1*).

Relative to the Lund validation cohort, the Malmö validation cohort had tumours that were more frequently detected based on symptomatic presentation (52 *versus* 42 per cent; *P* < 0.001) and that had high Ki-67 expression (54 *versus* 43 per cent; *P* = 0.023). Vascular invasion was more commonly observed in the Lund cohort, whereas the Malmö cohort had a higher proportion of HER2+ tumours.

Interestingly, both centres had high proportions of missing Ki-67 data; the Lund centre had a higher proportion of cases with missing vascular invasion data, and the Malmö centre had a higher proportion of cases with missing HER2 data. These differences might be explained by different registration routines at the two centres. Thus, a sensitivity analysis was performed by recoding the missing HER2 values as non-amplified to validate the predictive performance of the SUS nomogram. The HER2 sensitivity analysis did not substantially alter the nomogram’s predictive ability (data not shown).

### External validation of the SUS nomogram

For predicting a disease-free axilla (N0 *versus* N+), the original SUS nomogram had an AUC value of 0.74 (95 per cent c.i. 0.70 to 0.79) based on detailed clinicopathological data that were extracted from the medical records of patients who were diagnosed between January 2009 and December 2012 in Lund (development cohort, 598 patients). Internal validation using bootstrap datasets revealed slight optimism in that AUC, based on a minor decline in the discriminative performance (–0.009).

Using only routinely collected registry data, the AUC value for the same period was 0.76 (95 per cent c.i. 0.72 to 0.80) in the Lund cohort and 0.73 (95 per cent c.i. 0.70 to 0.76) in the Malmö cohort (*Table 2*). The AUC value for the time period 1 year before and 1 year after the SUS nomogram development period (i.e., 2008 and 2013) was 0.75 (95 per cent c.i. 0.70 to 0.81) in the Lund cohort and 0.74 (95 per cent c.i. 0.70 to 0.79) in the Malmö cohort. The average AUC value for the entire validation period (i.e., 2008–2013) was 0.75 (95 per cent c.i. 0.72 to 0.79) in the Lund cohort and 0.73 (95 per cent c.i. 0.71 to 0.76) in the Malmö cohort.

**Table 2:** Performance of the Skåne University Hospital nomogram for prediction a disease-free axilla *versus* any nodal metastasis in the development cohort and the validation cohort

Cohort	Time period	Data source	*n*	AUC
**Development cohort**				
Lund, original SUS nomogram	2009–2012	Medical records	598	0.74 (0.70–0.79)
**Validation cohort (all)**				
Lund and Malmö	2008–2013	NKBC data	2939	0.74 (0.72–0.77)
**Validation cohort (subgroups)**				
a) Lund	2009–2012	NKBC data	916	0.76 (0.72–0.80)
b) Malmö	2009–2012	NKBC data	1086	0.73 (0.70–0.76)
c) Lund	2008, 2013	NKBC data	402	0.75 (0.70–0.81)
d) Malmö	2008, 2013	NKBC data	535	0.74 (0.70–0.79)
e) Lund	2008–2013	NKBC data	1318	0.75 (0.72–0.79)
f) Malmö	2008–2013	NKBC data	1621	0.73 (0.71–0.76)

Values in parentheses are 95% confidence intervals. The validation cohort is stratified according to site (Lund and Malmö) and time period. **a** and **b**, overlapping the development period of the original Skåne University Hospital (SUS) nomogram (2009–2012). **c** and **d**, 1 year before and 1 year after the nomogram development period (2008 and 2013). **e** and **f**, the entire validation period (2008–2013).

AUC, area under the receiver operating characteristic curve;

NKBC, The National Quality Registry for Breast Cancer of Sweden.

**Table 3:** Possible sentinel lymph node biopsy reduction rates within the validation cohort using the Skåne University Hospital nomogram for predicting a disease-free axilla at cut-offs corresponding to maximized negative predictive value and false-negative rates of 5 and 10 per cent to reflect the acceptable false-negative rates for sentinel lymph node biopsy

	**N0 *versus* N+** **(*n* = 2939)**			
**Cut-off:** **Max NPV 0.95**	**TP**	**TN**	**FP**	**FN**
**No.**	**1007**	**19**	**1912**	**1**
**SLNB Reduction Rate**	(TN+FN)/(TP +TN +FP +FN) = 0.68%			
**False-Negative Rate**	FN/(TP+FN) = 0.10%			
**Cut-off: NPV 0.89**	**TP**	**TN**	**FP**	**FN**
**No.**	**957**	**396**	**1535**	**51**
**SLNB Reduction Rate**	(TN+FN)/(TP +TN +FP +FN) = 15.21%			
**False-negative rate**	FN/(TP+FN) = 5%			
**Cut-off: NPV 0.87**	**TP**	**TN**	**FP**	**FN**
**No.**	**907**	**658**	**1273**	**101**
**SLNB Reduction Rate**	(TN+FN)/(TP +TN +FP +FN) = 25.83%			
**False-Negative Rate**	FN/(TP+FN) = 10%			

Data on TP, TN, FP and FN are mean values from 200 imputed data sets rounded to the nearest integer.

N0, disease-free axilla; N+, any lymph node metastasis; SLNB, sentinel lymph node biopsy; Max NPV, maximum negative predictive value; TP, True positive; TN, True negative; FP, False positive; FN, False negative.

**Fig. 3:**
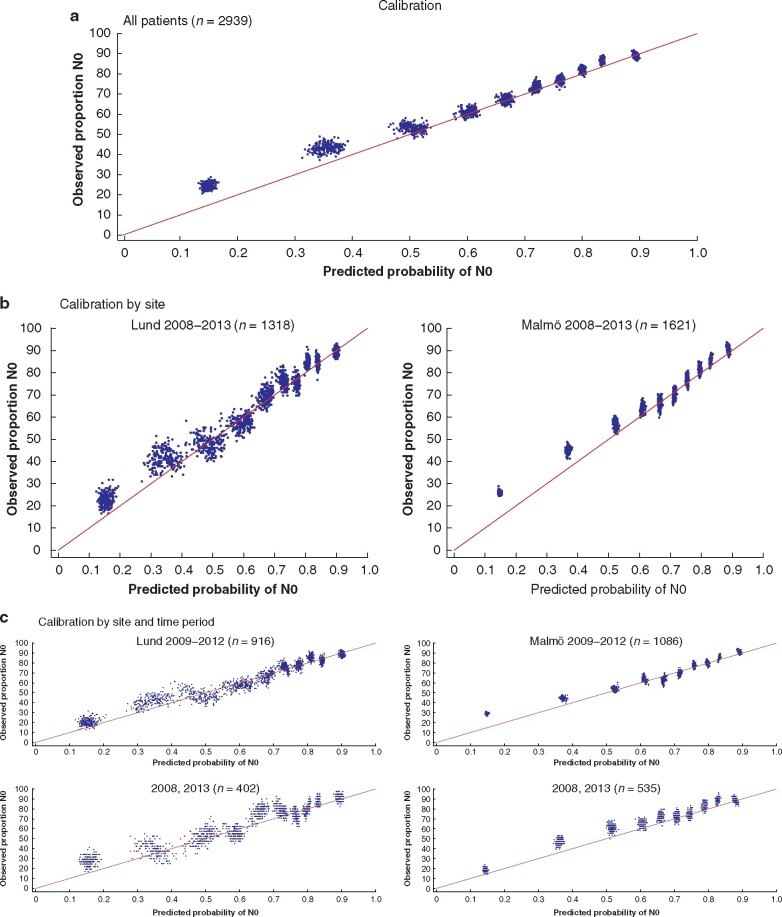
Calibration of the Skåne University Hospital nomogram according to site and time period The observed and predicted proportions of N0 disease in deciles based on the Skåne University Hospital (SUS) nomogram. Two hundred calibration plots (one per imputed dataset) were superimposed. **a** The entire validation period (Lund and Malmö, 2008–2013). Median Hosmer–Lemeshow *P* < 0.001 **b** Calibration stratified according to the breast centre site during the validation period (Lund 2008–2013(median Hosmer–Lemeshow *P* = 0.031) *versus* Malmö 2008–2013 (median Hosmer–Lemeshow *P* < 0.001)). **c** Calibration stratified according to the breast centre site and time period, with overlapping of the development period for the original SUS nomogram (Lund 2009–2012 (median Hosmer–Lemeshow *P* = 0.131) *versus* Malmö 2009–2012 (median Hosmer–Lemeshow *P* < 0.001)), as well as 1 year before and 1 year after the nomogram development period (Lund 2008 and 2013 (median Hosmer–Lemeshow *P* = 0.087) *versus* Malmö 2008 and 2013 (median Hosmer–Lemeshow *P* = 0.161)).

Calibration plots stratified according to site (Lund and Malmö) and time period revealed satisfactory agreement between the predicted probability of N0 and the observed proportion of patients with a disease-free axilla, especially among cases with a high probability of N0 (*Fig. 3*). However, the model overestimated the risk of nodal metastasis in patients with a high probability of N+ (low probability of N0), based on the lower-left cluster of dots above the 45° line for perfect calibration (*Fig. 3*). For the entire validation cohort (Lund and Malmö, 2008–2013), the Hosmer–Lemeshow test rejected the null hypothesis of perfect calibration with a median *P* value of <0.001 over the 200 imputations. Overlapping the development period of the original SUS nomogram (2009–2012), the test provided a *P* value of 0.131 for the Lund cohort and a *P* value of 0.001 for the Malmö cohort. The Hosmer–Lemeshow test also revealed a *P* value of 0.087 for the Lund cohort and a *P* value of 0.161 for the Malmö cohort when assessing the time periods 1 year before and 1 year after the SUS nomogram development period (2008 and 2013). For the entire validation period (2008–2013), the results were *P *=* *0.031 for Lund and *P *<* *0.001 for Malmö.

### Implications of the SUS nomogram for reduction in SLNB

**Fig. 4:**
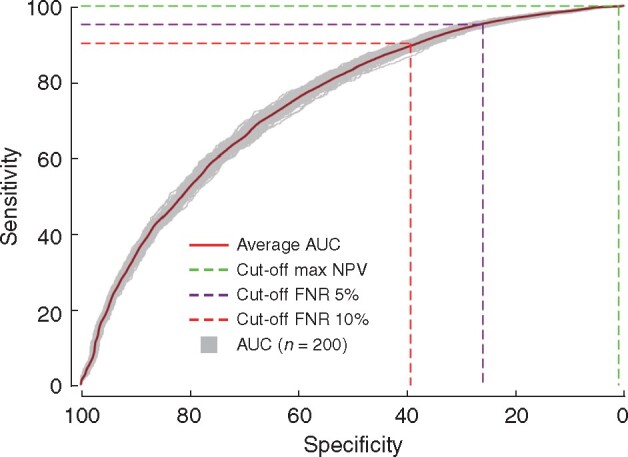
The discriminative performance of the Skåne University Hospital nomogram for predicting a disease-free axilla in the entire validation cohort Two hundred areas under the curves (AUC, one per imputed dataset) are shown, plus the average AUC, to illustrate the discriminative performance of the Skåne University Hospital nomogram. The cut-offs were maximized negative predictive value (Max NPV) and false-negative rates (FNR) of 5 and 10 per cent to reflect the acceptable FNR for sentinel lymph node biopsy. Minimum AUC, 0.726; mean AUC, 0.744; maximum AUC, 0.758.

An NPV-orientated cut-off was assessed to evaluate the ability of the SUS nomogram to identify patients with a very low probability of nodal metastasis, who might not benefit from axillary staging via SLNB (*Fig. 4*). The mean maximized NPV was 95 per cent (FNR of 0.10 per cent) (*Table 3*). If alternative maximum FNRs of 5 and 10 per cent were used as cut-offs, which reflect the accepted FNRs for the SLNB procedure, the possible SLNB reduction rates were in the range of 15–26 per cent (*Fig. 4* and *Table 3*).

## Discussion

This study validated the ability of the SUS nomogram to predict a disease-free axilla (N0 *versus* N+) in a non-selected population-based cohort of 2939 consecutive cases. The discriminative ability in the validation cohort (AUC: overall, 0.74; the Lund centre, 0.75; and the Malmö centre, 0.73), which only included routinely collected registry data, was similar to that of the original SUS nomogram in the development cohort (internally bootstrap-validated AUC: 0.74)^21^. In this context, models’ predictive performances tend to decline in validation sets because of overfitting to the derivation data, which is known as optimism. Although several internal validation techniques address this issue, external validation is necessary to address the generalizability of a model’s predictive ability^29^. In general, an AUC value of 0.70–0.80 is thought to confirm that a model has good predictive power^30^. Thus, the external validation findings (AUCs of 0.73–0.75), based on two centres with some differences in clinicopathological characteristics, indicate that the SUS nomogram is generalizable and may be applied to breast cancer patients from populations with similar characteristics. It may also be useful in populations with a different prevalence of lymph node negativity (64 per cent in the development cohort and 66 per cent in the validation cohort) and/or in cohorts with a different case mix. However, such applications would require recalibration of the predictor weights in the model.

A systematic review regarding the external validation of multivariable clinical prediction models concluded that calibration is an important measure of predictive performance that was missing in most studies^31^. This may at least partly explain the large number of published prediction models that have not been implemented in clinical practice. The present study revealed satisfactory calibration of the SUS nomogram, especially for patients with a low risk of nodal metastasis. Thus, the SUS nomogram may guide surgeons in counselling patients who are most likely to have a disease-free axilla and may be spared the SLNB procedure. However, the *P* value from the Hosmer–Lemeshow test revealed fairly strong evidence against perfect calibration, indicating that the SUS nomogram seems to overestimate the risk of nodal spread when the risk of nodal metastasis is high, which, although not satisfactory, indicates appropriate nomogram cautiousness. Unsurprisingly, the best discrimination and calibration was observed for the Lund cohort that was treated during the same time period as the development cohort. Analyses in this subcohort validated the nomogram’s performance using only routinely collected registry data. The truly external validations were based on patients who were not treated at Lund during 2009–2012, which provide validation at the same site but during another time period as well as validation at another site during the same or different time periods. The truly external validation subsets provided generally similar results, with AUCs of 0.73–0.75 and calibration plots that exhibited the same pattern.

Although ALN status remains an important prognostic factor for primary breast cancer, the necessity of extensive surgical axillary staging has been questioned during the last decade. The IBCSG 23-01^32^ and ACOSOG Z0011^33^ trials revealed that the omission of completion ALND was not inferior in terms of locoregional control or survival when the SLNB displayed limited metastasis in patients with a clinically node-negative axilla who underwent breast-conserving surgery, adjuvant breast irradiation and systemic treatment. Long-term follow-up of prospective randomized trials from the pre-SLNB era comparing ALND/axillary radiotherapy to no axillary surgery also revealed no significant difference in disease-free survival, although the rate of locoregional recurrence was somewhat increased when axillary surgery was not performed^34–37^.

The use of intensified adjuvant therapies over the last decade, which generally target tumour biological factors such as tumour molecular subtype, may further reduce the rates of axillary recurrence to 0.7–0.9 per cent after a benign SLNB result, as reported in early studies^38^^,^^39^. The present study evaluated whether the SUS nomogram could be used clinically to identify patients with a low risk of nodal metastasis who might benefit from the omission of surgical axillary staging, based on acknowledged FNR of 5–10 per cent for the SLNB technique. The results revealed possible SLNB reduction rates in the range of 15–26 per cent if FNR of 5–10 per cent were accepted for the prediction of disease-free axilla. Thus, the NPV-orientated cut-offs may be adjusted to guide surgeons in counselling patients regarding the omission of surgical axillary staging. The results are awaited of the randomized multicentric SOUND trial, which is comparing SLNB *versus* observation when the axillary ultrasound examination yields negative results^8^, and ultrasonographic axillary imaging with or without fine-needle biopsy remains a part of the routine preoperative work-up. Nevertheless, these imaging findings are influenced by the operator-dependent nature of axillary ultrasonography and the challenges of using imaging to determine accurately the nodal metastasis risk in low-burden disease. Therefore, the SUS nomogram may be an additional clinical tool for evaluating the nodal metastasis risk based on tumour biology, patient characteristics and the mode of detection. The SUS nomogram may provide additional information regarding axillary disease in this setting.

Since the publication of the Memorial Sloan-Kettering Cancer Center nomogram for estimating the risk of SLNB metastasis^16^, other nomograms have also been developed in more contemporary cohorts for predicting the risk of nodal metastasis^18^^,^^40^. However, the SUS nomogram is the first tool for predicting the likelihood of a disease-free axilla, for guiding the decision to omit axillary surgery for patients with the lowest likelihood of having nodal disease and to address potential SLNB reduction rates. Intrinsic tumour characteristics are known to be related to prognosis and locoregional control^41^^,^^42^, and the SUS nomogram incorporates the surrogate molecular subtypes to capture the lower risk of nodal metastasis for TNBC. Similarly, in the era of mammographic screening, the tumour detection mode also adds predictive value to the nomogram, alongside other key variables.

The most important limitation of the present study is the substantial proportion of missing data regarding some of the key pathological predictors in the validation cohort. This lack of completeness is a common problem in studies that use registry-based data sets. The predictors with the highest proportions of missing data were Ki-67 status and HER2 status, which are components of the surrogate molecular subtype, and vascular invasion. Multiple imputation was used to handle missing data, as this strategy provides unbiased estimates of missingness, and the missing values can thus be considered random, conditional on the other observed data, which is known as the missing at random assumption. This strategy has been shown to be effective even at high proportions of missing data^43^.

The highest true validation value (AUC: 0.75) suggests that the SUS nomogram does not perfectly predict a disease-free axilla, which highlights the complexity of lymphatic spread. Although more complex prediction models may have certain advantages for estimating nodal involvement^44^^,^^45^, the SUS nomogram is a readily available and user-friendly predictive tool in clinical settings. Studies based on retrospective registry data may be considered unreliable, given the risks of incomplete or improperly recorded data. Although the preoperative applicability of the original SUS nomogram depends on information obtained from histopathology results of core needle biopsy specimens, these validation results yielded the same AUC value. Thus, the nomogram’s utility was confirmed even with the use of only routine registry data with large amounts of missing data. The findings of this study are also strengthened by the use of a non-selected population-based validation cohort and the attempt to distinguish a disease-free axilla from any lymph node disease. The discrimination between node-negative cases and cases with any metastatic burden in the lymph nodes, including micrometastatic deposits, provides a more rigorous and cautious approach to risk estimation. Therefore, the SUS nomogram may help guide surgeons in counselling patients who are the least likely to have nodal metastasis and who may be safely spared from SLNB for axillary staging. Nevertheless, these results must be validated in other regions and patient populations to confirm that the SUS nomogram is universally applicable.

## Funding

The study was supported by grants from the Skåne County Councils Research and Developmental Foundation, the Governmental Funding of Clinical Research within the National Health Service (ALF), the Swedish Cancer Society, the Erling Persson Family Foundation, the Gunnar Nilsson Cancer Foundation, the Einar and Inga Nilsson Foundation, Malmö University Hospital Cancer Research Fund, Skåne University Hospital Funds and Donations and the Central Hospital of Kristianstad.

The funders played no role in the study design, data collection and analysis, decision to publish, or preparation of the manuscript.

## Notes

Preliminary findings were published as part of S.M.’s doctoral thesis.
